# Dissecting the Potential Roles of *Nigella sativa* and Its Constituent Thymoquinone on the Prevention and on the Progression of Alzheimer's Disease

**DOI:** 10.3389/fnagi.2018.00016

**Published:** 2018-02-09

**Authors:** Marco Cascella, Sabrina Bimonte, Antonio Barbieri, Vitale Del Vecchio, Maria Rosaria Muzio, Andrea Vitale, Giulio Benincasa, Anna Bella Ferriello, Amalia Azzariti, Claudio Arra, Arturo Cuomo

**Affiliations:** ^1^Division of Anesthesia and Pain Medicine, Istituto Nazionale Tumori, IRCCS Fondazione G. Pascale, Naples, Italy; ^2^S.S.D. Sperimentazione Animale, Istituto Nazionale Tumori, IRCCS Fondazione G. Pascale, Naples, Italy; ^3^Division of Infantile Neuropsychiatry, UOMI-Maternal and Infant Health, Naples, Italy; ^4^Pineta Grande Hospital, Caserta, Italy; ^5^Experimental Pharmacology Laboratory, IRCCS Istituto Tumori Giovanni Paolo II, Bari, Italy

**Keywords:** *Nigella sativa*, thymoquinone, natural compounds, Alzheimer's disease, oxidative stress

## Abstract

Several nutraceuticals have been investigated for preventing or retarding the progression of different neurodegenerative diseases, including Alzheimer's disease (AD). Because *Nigella sativa* (NS) and its isolated compound thymoquinone (TQ) have significant anti-oxidant and anti-inflammatory proprieties, they could represent effective neuroprotective agents. The purpose of this manuscript is to analyze and to recapitulate the results of *in vitro* and *in vivo* studies on the potential role of NS/TQ in AD's prevention and treatment. The level of evidence for each included animal study has been assessed by using a modified CAMARADES (Collaborative Approach to Meta-Analysis and Review of Animal Data from Experimental Studies) 10-item checklist. We used MEDLINE and EMBASE databases to screen relevant articles published up to July 2017. A manual search was also performed. The database search yielded 38 studies, of which 18 were included in this manuscript. Results from these approaches suggest that NS or TQ could represent an effective strategy against AD due to the balancing of oxidative processes and the binding to specific intracellular targets. The overall effects mainly regard the prevention of hippocampal pyramidal cell loss and the increased cognitive functions.

## Introduction

Although the knowledge of the exact pathophysiological mechanisms remains an unresolved issue, an emerging evidence underlines the role of the oxidative damage and microglia-mediated neuro-inflammatory responses in the initiation of neurodegenerative disorders including Alzheimer's disease (AD) (Mosher and Wyss-Coray, 2014). Experimental findings demonstrated that in AD pathogenesis, the 4.2-kD amyloid β peptide (Aβ)-dependent microglial activation leads to neuronal injury through a complex cascade by involving the secretion of various pro-inflammatory molecules such as tumor necrosis factor-α (TNF-α), interleukin (IL) IL-6, IL-1β, reactive oxygen species (ROS), and reactive nitrogen species (NOS) (Agostinho et al., 2010). In turn, the neuroinflammation and oxidative stress processes are responsible for the impairment of the neurovascular working leading to an axonal demyelination, local hypoxia–ischemia, and to restoring white matter damages (Iadecola, 2010).

Differently from a big amount of data relating to its pathogenesis, a poor number of AD therapeutics has been developed so far. Thus, many investigations have been performed to evaluate new neuroprotective agents. For instance, several clinical studies suggested that a high dietary antioxidant intake (e.g., vitamin E, flavonoids, and omega-3 fatty acids) could reduce the risk of AD (Andrade and Radhakrishnan, 2009). On the contrary, it has been reported that high dosage of B vitamin supplements (folate, B6, and B12) was not able to retard cognitive decline in patients with mild to moderate AD (Aisen et al., 2008). Moreover, in addition to the clinical investigations most experimental studies have been conducted *in vitro* or in animal models of AD to test natural compounds with antioxidants proprieties such as epigallocatechin gallate (EGCG) (Cascella et al., 2017a), quercetin (Schültke et al., 2003), kaempferol (Lei et al., 2012), resveratrol (Zhao et al., 2015) as potential protective factors to neurodegeneration induced by oxidative stress (Mecocci et al., 2014; Essa et al., 2016; De Domenico and Giudetti, 2017).

Furthermore, plant compounds and derivates could represent an important strategy for preventing or delaying the functional decline in AD's patients (Laver et al., 2016). For instance, *Ginkgo biloba* which shows rapid penetration and absorption into the brain is a well-studied tree in AD (Yuan et al., 2017), whereas curcumin reduced Aβ-related cerebral burden and neuroinflammation in transgenic AD mice (Lim et al., 2001). Thus, there is a great interest about the nutrient's role in the development of new preventive and therapeutic areas regarding AD and other neurodegenerative disorders (Poulose et al., 2017).

*Nigella sativa* (NS), also identified as black seed or black cumin, is a flowering plant belonging to the *Family Ranunculaceae*. In several countries such as the Middle East, Northern Africa and Southwest Asia, NS's seeds and its derivative, have been used not only as a spice and food preservative, but also as a protective and curative remedy for many disorders (Gholamnezhad et al., 2016; Hayatdavoudi et al., 2016). Chemically, NS contains 30% fixed oils (mainly fatty acids), 0.40–0.45% volatile oils, vitamins, amino acids, proteins, carbohydrates, alkaloids, saponins, crude fiber, and minerals. NS oil (NSO) is rich in polyunsaturated fatty acids (PUFA), phytosterols, TQ (up to 25% in volatile oil), carvacrol, t-anethole, sesquiterpenelongifolene, and 4 terpinol (Liu et al., 2011; Akram Khan and Afzal, 2016).

It is of note that TQ has anti-oxidant and anti-inflammatory proprieties (Ragheb et al., 2009). This compound, indeed, has an effect on free radical scavenging enhanced by many factors, such as its redox features and its ability to bypass the biological barriers and to move into subcellular compartments (Badary et al., 2003). Consequently, several investigations showed the antiasthmatic (Keyhanmanesh et al., 2010), antibacterial (Boudiaf et al., 2016), antidiabetic (Bamosa et al., 2010), hepatoprotective (Yildiz et al., 2008), antinociceptive (Abdel-Fattah et al., 2000), and antihypertensive (Jaarin et al., 2015) properties of NS and TQ. Furthermore, other researchers focused the studies on their anticancer activities (Kundu et al., 2014) as well as on their potential role in the prevention and/or treatment of toxic effects of anticancer drugs (e.g., chemotherapy-induced nephrotoxicity) (Cascella et al., 2017b). Important findings suggest that TQ can be considered an additive treatment option in neurological and neurosurgical practice (Elmaci and Altinoz, 2016). Detailed description on the protective roles of *N. sativa* and TQ in multiple disorders have been also described by Javidi et al. (2016), and Sahak et al. (2016). Moreover, the authors identified in *N. sativa* a potential candidate for the treatment of physiological and neurological disorders.

Recently, Bin Sayeed et al. (2013) showed that NS (500 mg capsule twice daily for 9 weeks) enhanced memory, attention, and cognition in healthy human volunteers. In another paper, the authors found that a reduced assumption of NS (500 mg once daily for 4 weeks) was enough to stabilize mood, decrease anxiety and modulate cognition positively in healthy adolescent males, compared to placebo (Bin Sayeed et al., 2014). The beneficial effects of NSO on learning and memory abilities were also confirmed in healthy male adult Sprague Dawley rats (Sahak et al., 2013).

In a light of these findings, our purpose is to analyze and to recapitulate the outcome of the pre-clinical studies on the potential roles of NS, and its constituent TQ, for preventing and slowing the AD's progression.

## Bibliografich research

This review was written according to Preferred Reporting Items for Systematic Reviews and Meta-Analyses (PRISMA) guidelines (Liberati et al., 2009). An implementation to the research was obtained by computer-operated search strategy using Medline and Embase databases up to July 2017. Two reviewers (M.C. and S.B.) engineered the review protocol, selected the inclusion and exclusion criteria and assessed the potential articles for the inclusion into the review. The search was performed by using the following terms to detect the specific nutraceutical (“thymoquinone” OR “*Nigella sativa*”) combined with the clinical condition (“Alzheimer's” OR “dementia”) or the term (“memory”). Additionally, was performed a manual search of the reference lists of identified articles. Inclusion criteria for study selection were as follows: performed in cell cultures, on animals, systematic reviews and meta-analysis on the topic. All genders and strains were included for animal studies. Published abstracts, summarizing studies, were excluded. Two independent reviewers (V.D.V. and A.B.) screened and assessed for inclusion, the articles identified by the search. The two reviewers agreed on 95% of the selected article and reached consensus on all included studies through collective discussions with a third reviewer (M.R.M.).

## Results

**Figure 1:**
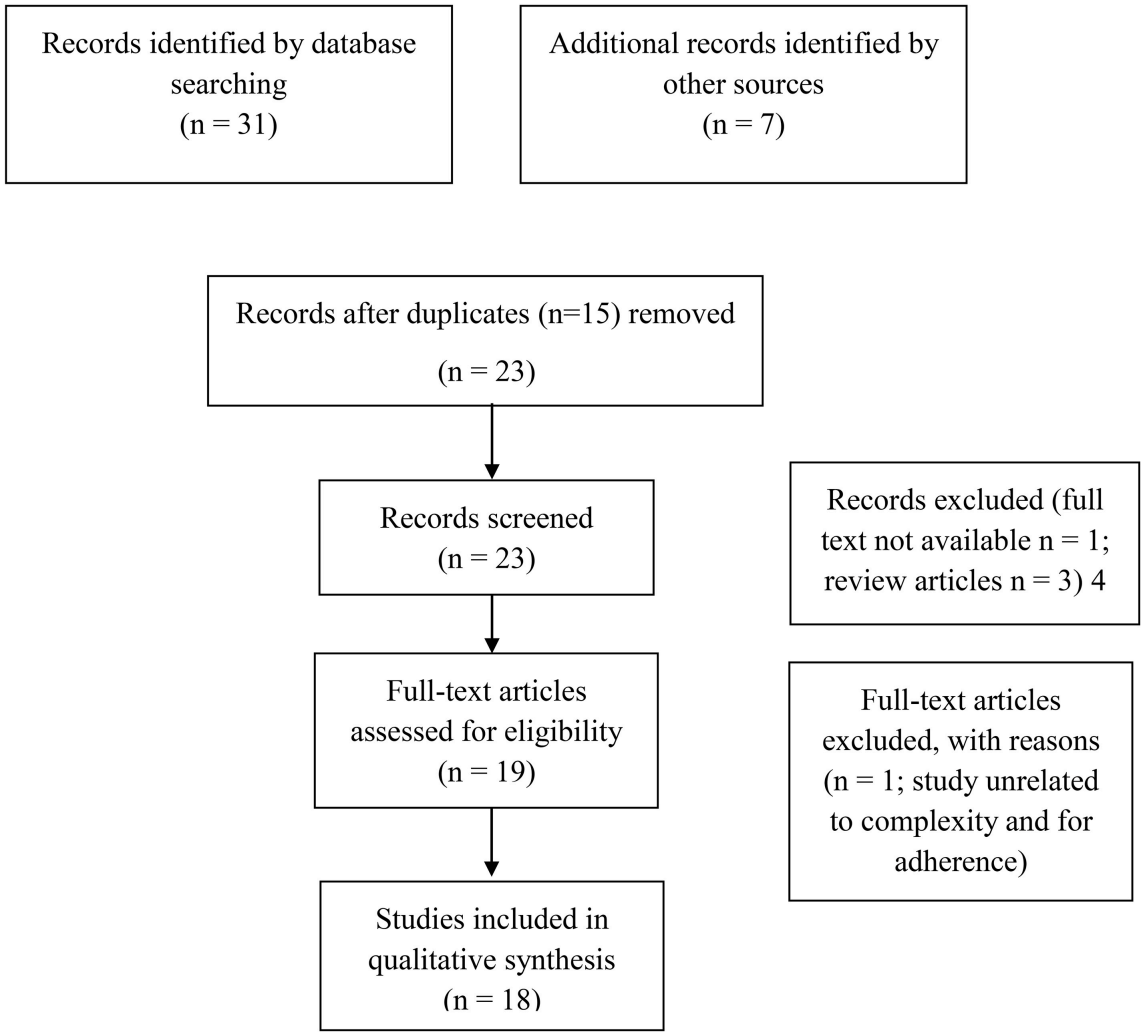
PRISMA flow diagram.

Firstly, the search strategy (showed in Figure 1) identified 31 articles. Thanks to the manual search, additional 7 records were identified and 15 duplicated were removed. Consequently, 23 non-duplicated articles were identified and full texts recovered. After the record screening, 4 articles were excluded (1 full text not available and 3 review articles). Of the remaining 19, 1 study was excluded for the reasons reported in the flow chart. Thus, 16 eligible studies were included in this review. For the narrative synthesis, we divided the available studies into two categories: *in vitro studies: an overview on the molecular mechanism underlying the anti-Alzheimer effects of NS or TQ, and Pre-clinical in vivo studies on the preventive effects of NS or TQ on AD*. The quality of evidence of each pre-clinical *in vivo* study was assessed by using a modified CAMARADES (Collaborative Approach to Meta-Analysis and Review of Animal Data from Experimental Studies) 10-item checklist (Macleod et al., 2004). Two authors (A.B.F. and A.V.) independently assessed the study's quality and any disagreements were solved through discussion or consultation with the corresponding author.

### *In vitro* studies: an overview on the molecular mechanism underlying the anti-alzheimer effects of NS or TQ

Several in *vitro* studies on the anti-oxidant and anti-neuroinflammatory properties of TQ have been conducted on different cells lines, including primary rat cerebellar granule neurons (CGNs), PC-12, E18, hi-PSC, SH-Y5Y, BV-2, and N2a cells as showed in Table 1. Evidences from these studies, strongly suggested that TQ has a great potential against neurotoxicity induced by Aß peptide which, in turn, plays a pivotal role in AD's pathogenesis.

**Table 1:** *In vitro* studies on the effects of NS or TQ in AD.

**Cell lines/*in vitro* test**	**Drugs and dosages**	**Main results**	**References**
Dried plant /AChE inhibition assay	Galanthamine; Thymohydro-quinone Carvacrol; Thymol; Linalool. (10-0,00001 mg/mL)	AChE inhibitory potential decreased as follows: Galanthamine > Thymohydro-quinone > Carvacrol > TQ > Total essential oil > Thymol > Linalool.	Jukic et al., 2007
Primary rat CGNs	NSO (500–2 mg/mL)	NSO and its fractions prevented the Aβ toxicity (NSO, and WF more than HF, and EAFs).	Ismail et al., 2008
PC-12	TQ (0.78–400 μM)	TQ: (1) ameliorated induced loss of cell viability; (2) prevented the Aß25–35-induced increase activity of LDH; (3) had protective effects on GPx, GR, and AChE in PC 12 cells exposed to Aß25–35; (4) downregulated the iNOS expression along with NO level; (5) had a protective role of intracellular oxidative stress, by restoring the ROS level; (6) augmented the membrane potential by restoring the normal level of MMPs.	Khan et al., 2012
E18	TQ (0.1, 1, 10, 100 nM)	TQ: (1) reduced intracellular ROS level in neurons treated with Aß1–42; (2) reduced Aß-induced inhibition of synaptic vesicle recycling.	Alhebshi et al., 2013
CGNs	TQ (0.1 and 1 μM)	TQ had protective effects on Aß1–40-induced neurotoxicity by reducing the production of free radical on Aβ1–40 in CGNs and by attenuating the activation of Caspase-3,−8, and−9 on Aß1–40 exposure.	Ismail et al., 2013
Rat primary hippocampal cells; hiPSC-derived neurons	TQ (100 nM)	TQ: (1) neurons were protected against SN-induced synapse damage and the synaptophysin was enhanced; (2) maintained the synaptic activities in hippocampal neurons were maintained; (3) the uptake of FM1-43 dye increased, while the inhibitory effect of SN on synaptic vesicle recycling was decreased.	Alhebshi et al., 2014
SH-SY5Y	TQ, EGCG; DLPC	TQ prevented the oxidation of Aß by decreasing the expression of NO and by increasing the GSH levels.	Kennedy et al., 2014
BV-2	TQ (0–40 μM)	TQ treatment in the LPS/IFNγ-activated microglia altered the expression profiles of Ccl5, Nos and Ptgs2.	Cobourne-Duval et al., 2016
N2a	NOS encapsulated in Nanoparticles-pDNA (ratio from 5 to 50%)	Encapsulated NSO promoted neurite outgrowth of N2a cells.	Doolaanea et al., 2016

*AChE, acetylcholinesterase; NSO, Nigella Sativa Oil; TQ, Thymoquinone; CGNs, cerebellar granule neurons; HF, hexane fraction; EAF, ethyl acetate fraction; WF, water fraction; GPx, glutathione peroxidase; GR, glutathione reductase; Aß, amyloid beta; iNOS, Inducible nitric oxide synthase; NOS, nitric oxide synthase; ROS, Reactive oxygen species; MMPs, Matrix metalloproteinases; ß-amyloid peptide 1–40 sequence, Aβ1–4; human induced pluripotent stem cells (hiPSC); α-synuclein, αSN; (FM1-43 dye, N-(3-Triethylammoniumpropyl)-4-(4-(Dibutylamino) Styryl) Pyridinium Dibromide); NO, nitrix oxide; GSH, glutathione; LPS, lipopolysaccaride; IFNγ, Interferon-gamma; Ccl5, Chemokine (C-C) motif ligand; Nos2, nitric oxide synthase 2 inducible; Ptgs2, Pros-taglandin-endoperoxide synthase 2; Txnip, Thioredoxin-interacting protein; Prdx 1, Peroxiredoxin 1; S. cerevisiae, Sulfiredoxin 1 homolog*.

Jukic et al. (2007) reported that the principal constituents of essential oil *Thymus vulgaris*, thymol and carvacrol, and their derivatives (e.g., TQ and thymohydroquinone) had inhibitory effects on acetylcholinesterase (AChE) thanks to their anti-oxidant properties. Thus, indicating that these compounds can be identified as potential therapeutic options for the treatment of AD and/or of cognitive disorders. Ismail et al. (2008) reported findings on the neuroprotective role of NSO and its fractions [(e.g., hexane fraction (HF), ethyl acetate fraction (EAF) and water fraction (WF)] against Aß-induced cell death in primary rat CGNs. In these *in vitro* investigations, was reported that WF and NSO had a better protective effect on Aβ-induced toxicity in CGNs than HF and EAFs fractions, by acting on the antioxidant pathway. Khan et al. (2012), showed that TQ was able to reduce the neurotoxicity induced by Aß in an *in vitro* model of undifferentiated pheochromocytoma rat cell line, PC-12, by inhibiting the oxidative stress and the mitochondrial dysfunction through the reestablishment of the abnormal levels of Matrix metalloproteinases (MMPs) and ROS. These data confirmed the hypothesis that TQ, though the mechanism of neuroprotection, may be potentially used in the management of neurodegenerative disorders, including AD. Similar findings were obtained by a study conducted by Alhebshi et al. (2013). The authors proved that TQ played a role in the neuroprotection against neurotoxicity induced by Aß in rat primary neurons, E18, through the inhibition of ROS formation, and mitochondrial membrane depolarization. Moreover, they showed that TQ reduced Aß-induced inhibition of synaptic vesicle recycling. The neuroprotective properties of TQ in AD were also confirmed by a study conducted on CGNs cells exposed Aβ1–40 sequence (Aβ1–40) neurotoxicity (Ismail et al., 2013). Authors showed that the pre-treatment with TQ (0.1 and 1 μM) was able to prevent the generation of free radicals, and the apoptosis via caspase pathways, thus resulting in an improvement of cell viability. Additionally, TQ preserved the extensive neurite network and the morphology of cell bodies on Ab1–40-induced morphological damages in CGNs. Subsequently, Alhebshi et al. described a neuroprotective role of TQ and its potential application in patients with Parkinson's disease and dementia with Lewy bodies. Specifically, it has been showed that TQ protected cultured rat primary hippocampal and human induced pluripotent stem cells (hiPSC)-derived neurons against α-synuclein (αSN)-induced synaptic toxicity, by acting on synaptic vesicle recycling (Alhebshi et al., 2014). Interesting findings on the role of TQ as a potential preventive agent for AD's treatment, were also reported for human neuroblastoma cells, SH-SY5Y by Kennedy et al. (2014). To this regard, the authors demonstrated that TQ and other antioxidants [e.g., EGCG, dilinoleoylphosphatidylcholine (DLPC)] were able to prevent oxidation of Aß, by acting on TNF-α-mediated signaling pathway, through the downregulation of nitric oxide (NO) and the upregulation of glutathione (GSH). Recently (Cobourne-Duval et al., 2016), it has been demonstrated that TQ had antioxidant effects in activated BV-2 murine microglial cells, by acting on ROS and pro-inflammatory cytokines levels whose expression are extremely higher in several neurodegenerative disorders, including AD. Basically, the authors showed that the treatment with TQ of the lipopolisaccaride (LPS)/Interferon-gamma (IFNγ) activated microglia, impaired the expression profiles of specific genes involved in the oxidation process. Specifically, TQ was able to downregulate the expression of Chemokine (C-C) motif ligand 5 (Ccl5), nitric oxide synthase 2, inducible (Nos2), Prostaglandin-endoperoxide synthase 2 (Ptgs2), Thioredoxin-interacting protein (Txnip) and to upregulate the expression of Peroxiredoxin 1 (Prdx 1) and Sulfiredoxin 1 homolog (*S. cerevisiae*) (Sxrn1). Interestingly, by using an innovative technology based on the application of Poly Lactic-co-Glycolic Acid (PLGA) nanoparticles, Doolaanea et al. co-encapsulated NSO and plasmid DNA (pDNA) with PLGA nanoparticles in order to enhance gene therapy of AD's syndrome. The authors demonstrated that the encapsulated NSO promoted the outgrowth of murine neuroblastoma cells BV-2, thus resulting in a better efficacy for AD's treatment (Doolaanea et al., 2016).

Taken together, these findings strongly suggest that NS, or its constituent TQ, could represent an effective therapeutic agent with neuroprotective properties against AD, due to the action on different molecular pathways involved in its pathogenesis. Despite these encouraging results, more studies will be needed to translate these pieces of knowledge into clinical practice.

### Pre-clinical *in vivo* studies on preventive effects of NS or TQ on AD

The neuroprotective effects of NS and/or its constituent TQ have been also demonstrated by *in vivo* experiments as summarized in Table 2. Several experiments were performed on rat models of global cerebrovascular hypo perfusion, leading to hippocampus neurodegeneration and cognitive defects. The first study was reported by Al-Majed et al. (2006) in a rat model of transient forebrain ischemia-induced neural damage. Results from this study showed that TQ pretreatment significantly reduced the loss of hippocampal neuronal cells (*P* < 0.001), in the CA1 region. This effect was correlated with antioxidant activities of TQ on the levels of malondialdehyde, GSH, catalase, and on the superoxide dismutase (SOD) activities. A similar rat model was used by Hosseinzadeh et al. (2007). It was demonstrated that TQ and NSO had significant neuroprotective effect on hippocampal cells of rats, mainly correlated with the inhibition of lipid peroxidation after cerebral ischemia.

**Table 2:** *In vivo* experiments on the anti-neurodegenerative properties of NS or TQ in AD.

**Animal models**	**Methods**	**Tests**	**Results**	**References**
Wister-albino rats with ischemia-reperfusion injury (IRI) in hippocampus	TQ (5 mg/kg/day p.o.) 5 days before ischemia and continued during the reperfusion time.	Histological and Histochemical studies	TQ pre-treatment significantly attenuated the loss of hippocampal neuronal cells (*P* < 0.001), reduced the malondialdehyde's level and increased GSH, catalase and SOD activities.	Al-Majed et al., 2006
Sprague Dawley rats underwent to 2VO surgery	NSO (1 mg/kg), OG daily for 10 days prior to 2VO surgery and then for 70 days post 2VO surgery.	Memory tests	NSO had a protective effect on spatial cognitive functions.	Hosseinzadeh et al., 2007
Wistar rats with IRI	NS (ip 1mg/kg, 10mg/kg and 50 mg/kg), during the carotid clamp and after 72 h.	histopathological examinations	Prevention of intracellular edema of hippocampal interneurons and astrocytes with the highest dose (50 mg/kg).	Hobbenaghi et al., 2014
Wistar rats received IP injection of Aβ-25-35 (1 μl into the CA1 region) or scopolamine (IP 1 mg/kg)	Thymol (0.5, 1, or 2 mg/kg); Carvacrol (0.5, 1, or 2 mg/kg) IP injected 30 min before MWM.	Memory test; Acute toxicity	Thymol and carvacrol improved cognitive functions and reversed the effect of Aβ and scopolamine.	Azzubaidi et al., 2012
Rats with experimentally induced AD (LPS)	TQ (10 mg/kg IP), or nAChR agonist, plus PAM (for 5 days).	Histological and Histochemical studies	TQ, or a α7 nAChR agonist, in combination with PAM, attenuates neuroinflammation and activates MSCs.	Azizi et al., 2012
Streptozotocin-Induced Diabetic Rats	NSO (2 ml/Kg orally), MT (100 mg/Kg), GI (0.8 mg/Kg), and the insulin receptor inhibitor IOMe-AG538 (21 days).	Serum biochemical assays; Neuroinflammation cytokines profile; Brain oxidant and antioxidant markers expression; Cholinergic function; AGEs and brain insulin resistance. Modification of brain AD-related miRNA expression profile, were observed	NSO and the anti-diabetic drugs alone and/or in combination suppressed the oxidative stress, the amyloidogenic pathway and the pro-inflammatory mediator. A reduction in the insulin receptor inhibitory effect of IOMe-AG538 and a modification of the insulin-signaling pathway.	Balbaa et al., 2016, 2017
Rats with memory deficits induced by LPS.	2, 5, or 10 mg/kg TQ extract 30 min before IP LPS	Behavioral tests (PA, MWM); Biochemical measurements in hippocampal and cortical tissues	TQ was able to enhance memory impairments by reducing the hippocampal cytokine levels and brain's damage.	Bargi et al., 2017
Sprague Dawley rats fed with a high fat-cholesterol diet	TQ rich-fraction nanoemulsion (TQRFNE); TQ emulsion; TQ nanoemulsion.	Memory test (MWM); Serum antioxidant status; Genes expression levels in brain cortex and hippocampus	TQRFNE ameliorated behavioral changes, lipid peroxidation and soluble Aβ levels. Improved radical scavenging activity and increased antioxidants genes expression levels.	Ismail et al., 2017

*2VO, bilateral carotid arteries occlusion; NSO, Nigella Sativa oil; OG, oral gavage; IIP, intrahppocampal injection; IP, intraperitoneal injection; MWM, Morris water maze; AD, Alzheimer's Disease; Ischemia-reperfusion injury (IRI); TBRAS, thiobarbituric acid reactive substances; MDA, malondialdehyde; LPS, lipopolysaccharide; MSCs, mesenchymal stem cells; PAM, positive allosteric modulator (of nAChR); MT, Metformin; GI, glimepiride; TNF-α, tumor necrosis factor-α; MT, metformin; GI, glimepiride; AGEs, advanced glycation end products; PA, passive avoidance test; TQRFNE, TQ rich-fraction nanoemulsion*.

More recently, Hobbenaghi et al. (2014) by administrating NS in rats during the carotid clamp, showed that the strongest neuroprotective effect of NS, identified as the prevention of intracellular edema of hippocampal interneurons and astrocytes, was obtained with the highest dose (50 mg/kg). Later on, Azzubaidi et al. (2012) conducted behavioral investigations of animals by using the two-vessel occlusion (2VO) murine model. This model is obtained by a ligation and a cutting of the common carotid arteries, thus representing an optimal strategy to prove the so-called vascular hypothesis of AD responsible for neurodegeneration. In these experiments, long-term memory, short-term memory and working memory were investigated through the Morris water maze (MWM). Results showed that the oral administration of NSO (1 mg/kg) for 10 days prior to 2VO surgery and then for 70 days post 2VO surgery, highly preserved the spatial cognitive functions with an overall memory's and learning's improvements.

Azizi et al. (2012) reported an interesting study on the efficacy of Thymol and cravacrol, monoterpenic phenols also present in NS, against cognitive deficits in rat model of AD. Results suggested that the reduction of cognitive defects induced by treatment with thymol and carvacrol, was probably associated to their antioxidant and anti-inflammatory properties. Moreover, a lower toxicity of both compounds was also reported.

Recently, in an animal model of AD [lipopolysaccharide(LPS)-induced neuroinflammation in rats] it was tested the combination of PNU-120596, a positive allosteric modulator (PAM) of α7 nicotine acetyl choline receptor (nAChR), with the specific nAChR agonist PNU-282987 or with TQ (Ibrahim AbdEl Fattah et al., 2016). Data reported that TQ or the α7 nAChR agonist in combination with the PAM had a pivotal role in the treatment of AD, by reducing the neuroinflammation, and by activating the mesenchymal stem cells (MSCs). Indeed, by molecular [anti-phospho-cyclic adenosine phosphate (cAMP) response element binding protein (P-CREB), and CD44 Ab], and morphometric analyses (evaluation of the area of deformed neurons, and Aβ plaques) were reported a reduced number of deformed neurons and glial cells as well as an increased number of pyramidal cells. Interestingly, the combination of TQ, or α7 nAChR agonist, with PAM had a better effect in comparison with TQ alone.

A possible role of the brain insulin resistance and a reduced insulin signaling in the AD's pathogenesis, was also described by several studies (Aliev et al., 2014). Baalba et al., by using streptozotocin-induced diabetic (STZ-T2DM) rats, showed that the daily intake of NSO induced the expression of insulin receptor and altered the expression of insulin-like growth factor-1 and phosphoinositide-3 kinase (Balbaa et al., 2016). Based on these results, subsequently, the authors showed that in STZ-T2DM rats, NSO (2.0 ml/Kg) and anti-diabetic drugs (metformin and glimepiride) alone and/or in combination, were able to suppress brain levels of oxidative stress markers such as xanthine oxidase (XO) and NOS, through the product of lipid peroxidation [e.g., thiobarbituric acid reactive substances (TBARS)] (Balbaa et al., 2017). On the contrary, an increased activity of antioxidant agents (e.g., glutathione) and enzymes such as glutathione peroxidase (GPx), SOD and glutathione S-transferase (GST), was observed. Furthermore, the NSO administration lowered the insulin receptor inhibitory effect of IOMe-AG538 by modifying the insulin-signaling pathway and by reducing the levels of advanced glycation end products (AGEs) which are proteins or lipids involved in aging and in the pathogenesis of degenerative diseases, including AD. Again, the authors investigated on the modification of brain AD-related miRNA expression profile and demonstrated that NSO restored the brain tissue levels and serum values of miRNA, thus suggesting a possible role of miRNAs for the early diagnosis of AD.

Bargi et al. (2017), reported that TQ was able, yet at lower doses, to improve memory impairments induced by LPS in rats. These behavioral results were associated with a reduction of the hippocampal neuroinflammatory cytokines TNF-α, and IL6, a decreased level of markers of oxidative damage in brain tissues such as NOS, malondialdehyde (MDA) as well as an increased activity of SOD and catalase in hippocampus and cortex. Finally, in a recent paper Ismail et al. (2017) tested TQ rich-fraction nanoemulsion (TQRFNE) compared to TQ conventional emulsion and TQ nanoemulsion in rats fed with a diet rich of high fat-cholesterol for 6 months. The results were very interesting as TQRFNE ameliorated behavioral changes, lipid peroxidation and soluble Aβ levels. Furthermore, there were an improved radical scavenging activity and increased antioxidants genes expression levels in the brain cortex and in the hippocampus. These findings offer a further evidence that the presence of different compounds in the preparation (e.g., camphor, carvone, limonene, and thymol) may increase the effect of TQ alone. Moreover, the nanoemulsion may enhance the oral bioavailability and brain delivery.

Overall these results strongly suggest that NS, and its constituent TQ, show interesting proprieties mainly attributable to the balancing of the oxidative processes, whose overt activation represents a pivotal basis of neuroinfiammation and neurodegeneration.

According to the CAMARADES analysis (Table 3), although all the data obtained a good score (>5), we underline a severe gap in all the studies, due to the lacks of details for the allocation concealment. The Al-Majed's study (Al-Majed et al., 2006) presented the highest score (8/10) and it is the only research performed with a blinded assessment of outcome. In four studies the experimental protocols were drawn by combining the biochemical and histological evaluations. Other studies, focused mainly on the memory/behavioral tests such as the passive avoidance test and the classical MWM. Thus, not all the adopted criteria are always applicable.

**Table 3:** The quality of evidence obtained by using a modified CAMARADES (Macleod et al., 2004).

**Criteria**	**References**								
	**(Al-Majed et al., 2006)**	**(Azzubaidi et al., 2012)**	**(Hosseinzadeh et al., 2007)**	**(Hobbenaghi et al., 2014)**	**(Azizi et al., 2012)**	**(Ibrahim AbdEl Fattah et al., 2016)**	**(Balbaa et al., 2016)**	**(Bargi et al., 2017)**	**(Ismail et al., 2017)**
Publication in a peer-reviewed journal	√	√	√	√	√	√	√	√	√
Number of experiments and control groups report	√	√	√	√	√	√	√	√	√
Housing and Husbandry Conditions	√	√	√	√	√	√	√	√	√
Details of intervention/exposure group procedures	√	√	√	√	√	√	√	√	√
Random allocation to groups									
Allocation concealment									
Blinded assessment of outcome	√	NA							
Biochemical evaluations	√		√				√	√	√
Histological evaluations	√			√		√	√		
Statistical analysis	√	√	√	√	√	√	√	√	√
Total	8	5	6	6	5	6	7	6	6

*√, the criterion is satisfied; NA, not applicable*.

## Future research needs and translational perspectives in clinical practice

Although experimental studies on the protective role of NS/TQ and other nutraceuticals in AD report interesting findings, further investigations are needed in order to understand the molecular basis underlying the neuroinflammation and the neurodegenerative processes involved in AD's pathogenesis. An interesting issue concerns the mechanisms responsible for the prolonged activation of the classically activated proinflammatory microglia phenotype 1 (M1) as well as the transition between the M1 phenotype and the alternatively activated phenotypes 2 (M2) which promotes the tissue repairing through the action of anti-inflammatory cytokines (e.g., IL-10, IL-4, IL-3) (Wang et al., 2015). Since a recent study addressed the topic investigating on the flavonoid rutin (Bispo da Silva et al., 2017), it could be interesting to investigate the potential role of NS/TQ in different phases of microglia activation/transaction.

Further research are also needed in order to identify specific cellular and molecular targets. As a consequence, starting from this knowledge, it would be useful to design clinical trials by using several combinations of different compounds, with the scope of engineering an effective translational perspective into clinical practice. Interestingly, the combination of different antioxidants has already been tested successfully and it has been proved that there is a real association between the synergism of combined antioxidants. For instance, Zandi et al. (2004) demonstrated that despite the combination of vitamins E, B, and C supplements significantly reduced the risk of AD, the intake of antioxidant vitamins used alone did not reduce the risk of AD prevalence.

Another strategy of treatment could involve the association between an antioxidant (e.g., NS/TQ) and an agent of targeted therapy. For instance, Kitazawa et al. (2011) blocked in 3xTg-AD mice the IL-1 signaling and demonstrated a reduced neuroinflammation (e.g., nuclear factor kappa-light-chain-enhancer of activated B cells, NF-kB, activity), Aβ deposition and tau pathology. Moreover, other investigations have been conducted by blocking TNF signaling with TNF-α monoclonal antibody Infliximab (Shi et al., 2011) or in TNF knock-down mice, although the results were mixed (Montgomery et al., 2011). As alternative target for the treatment of AD's syndrome an interesting paper by Ettchetto et al. showed promising findings on the use of dexibuprofen in a mouse model of familial AD, by reducing the neuroinflammatory response, the levels of amyloid plaques and neurofibrillary tangles, while enhancing the memory (Ettcheto et al., 2017).

The preclinical research in this field has the difficult task of offering effective therapeutic opportunities to the clinical investigation whereas, to date, the role of nutraceuticals in AD prevention and treatment is of a limited clinical importance. For example, the British Association for Psychopharmacology stated that further evidence is needed to recommend the use of vitamin E and nutritional supplements for this purpose (O'Brien et al., 2017). More focused preclinical investigation on the role of nutraceuticals in neurodegenerative diseases could bypass these difficulties and thus could provide the evidence requested by the scientific community.

## Conclusion

To date, there is no drug identifiable as a gold standard in the AD's prevention or treatment. To cover this gap, many neuroprotective agents including nutraceuticals have been evaluated for their potential benefits. This review suggests that NS/TQ could have a significant role for preventing and retarding the progression of AD. The promising results of *in vitro* and *in vivo* preclinical investigations, and the safety profile of the compounds, should encourage further preclinical investigations and the translation into clinical practice through the drawing of long-term studies conducted on large population size of AD's patients. In particular, further investigations should be performed to elucidate the effectiveness of NS and its individual components. Indeed, despite TQ showed a better nephro-protective effect, no combinatorial studies on NS and its single compounds, have been performed. Moreover, the pharmacokinetics of these compounds, should be elucidated, since data about the brain's bioavailability and transport across blood-brain barrier, are still inconsistent. More pre-clinical studies will be extremely needed in order to highlight the potential interactions of NS/TQ with other drugs, to elaborate the long-term results and, finally to validate and compare the effectiveness of NS/TQ at different stages of AD.

## Author contributions

MC, SB and AB: written the review protocol, scheduled the inclusion and the exclusion criteria and assessed potential articles for inclusion into the manuscript; MM, AV, AF and VD: screened and assessed the articles; AF and AB: independently assessed study quality for the CAMARADES checklist; AB, AC, CA and AA: assisted all the reviewers; AV, AF, GB, CA: revised the tables. AA and GB: revised the English language. All authors partecipate to draft the work, to revise it critically for intellectual contents and approved the final version of manuscript.

### Conflict of interest statement

The authors declare that the research was conducted in the absence of any commercial or financial relationships that could be construed as a potential conflict of interest.

## Abbreviations

AD: Alzheimer's disease
Aβ: amyloid β peptide
TNF-α: tumor necrosis factor-α
IL: interleukin
ROS: reactive oxygen species
NOS: reactive nitrogen species
EGCG: epigallocatechin gallate
NS: *Nigella sativa*
PUFA: polyunsaturated fatty acids
TQ: thymoquinone
PRISMA: Preferred Reporting Items for Systematic Reviews and Meta-Analyses
CAMARADES: Collaborative Approach to Meta-Analysis and Review of Animal Data from Experimental Studies
CGNs: cerebellar granule neurons
AChE: acetylcholinesterase
NOS: *Nigella sativa oil*
HF: hexane fraction
EAF: ethyl acetate fraction
WF: water fraction
LDH: lactate dehydrogenase
iNOS: inducible nitric oxide synthase
MMPs: matrix metalloproteinases
hiPSC: human induced pluripotent stem cells
αSN: α-synuclein
FM1-43 dye: *N*-(3-Triethylammoniumpropyl)-4-(4-(Dibutylamino) Styryl) Pyridinium Dibromide
IFNγ: Interferon-gamma
Ccl5: chemokine (C-C) motif ligand
Nos2: nitric oxide synthase 2 inducible
Ptgs2: pros-taglandin-endoperoxide synthase 2
Txnip: thioredoxin-interacting protein
Peroxiredoxin 1: Prdx 1
Sxrn1: sulfiredoxin 1 homolog (*S. cerevisiae*)
PGLA: Poly Lactic-co-Glycolic Acid
pDNA: plasmid DNA
2VO: bilateral carotid arteries occlusion
IP: intraperitoneal
IIP: intraippocampal
IRI: ischemia-reperfusion injury
TBRAS: thiobarbituric acid reactive substances
MDA: malondialdehyde
LPS: lipopolysaccharide
PAM: positive allosteric modulator
MT: metformin
GI: glimepiride
nAChR: nicotin acetyl choline receptor
MSCs: mesenchymal stem cells
STZ-T2DM: streptozotocin-induced diabetes
XO: xanthine oxidase
GPx: glutathione peroxidase
SOD: superoxide dismutase
GST: glutathione S-transferase
AGEs: advanced glycation end products
M1: microglia phenotype 1
M2: microglia phenotype 2
NF-kB: nuclear factor kappa-light-chain-enhancer of activated B cells.
